# MicroRNA‐199a‐5p aggravates angiotensin II–induced vascular smooth muscle cell senescence by targeting Sirtuin‐1 in abdominal aortic aneurysm

**DOI:** 10.1111/jcmm.16485

**Published:** 2021-06-15

**Authors:** Wuyuan Tao, Yimei Hong, Haiwei He, Qian Han, Mengmeng Mao, Bei Hu, Hao Zhang, Xiaoran Huang, Wei You, Xiaoting Liang, Yuelin Zhang, Xin Li

**Affiliations:** ^1^ The Second School of Clinical Medicine Southern Medical University Guangzhou China; ^2^ Department of Emergency Medicine Department of Emergency and Critical Care Medicine Guangdong Provincial People's Hospital Guangdong Academy of Medical Sciences Guangzhou China; ^3^ Department of Medicine State Key Laboratory of Respiratory Disease The First Affiliated Hospital of Guangzhou Medical University Guangzhou Institute of Respiratory Health Guangzhou China; ^4^ School of Pharmacy Bengbu Medical College Bengbu China; ^5^ Clinical Translational Medical Research Center Shanghai East Hospital Tongji University School of Medicine Shanghai China

**Keywords:** abdominal aortic aneurysms, miR‐199a‐5p, senescence, Sirtuin1, vascular smooth muscle cells

## Abstract

Vascular smooth muscle cells (VSMCs) senescence contributes to abdominal aortic aneurysm (AAA) formation although the underlying mechanisms remain unclear. This study aimed to investigate the role of miR‐199a‐5p in regulating VSMC senescence in AAA. VSMC senescence was determined by a senescence‐associated β‐galactosidase (SA‐β‐gal) assay. RT‐PCR and Western blotting were performed to measure miRNA and protein level, respectively. The generation of reactive oxygen species (ROS) was evaluated by H2DCFDA staining. Dual‐luciferase reporter assay was used to validate the target gene of miR‐199a‐5p. VSMCs exhibited increased senescence in AAA tissue relative to healthy aortic tissue from control donors. Compared with VSMCs isolated from control donors (control‐VSMCs), those derived from patients with AAA (AAA‐VSMCs) exhibited increased cellular senescence and ROS production. Angiotensin II (Ang II) induced VSMC senescence by promoting ROS generation. The level of miR‐199a‐5p expression was upregulated in the plasma from AAA patients and Ang II–treated VSMCs. Mechanistically, Ang II treatment significantly elevated miR‐199a‐5p level, thereby stimulating ROS generation by repressing Sirt1 and consequent VSMC senescence. Nevertheless, Ang II–induced VSMC senescence was partially attenuated by a miR‐199a‐5p inhibitor or Sirt1 activator. Our study revealed that miR‐199a‐5p aggravates Ang II–induced VSMC senescence by targeting Sirt1 and that miR‐199a‐5p is a potential therapeutic target for AAA.

## 1 INTRODUCTION

Abdominal aortic aneurysm (AAA), characterized by degradation of the aortic wall and a progressively enlarged aorta that exceeds the normal diameter by > 50%, is a leading cause of morbidity and mortality worldwide.^1^ It is an age‐related vascular disease with an incidence as high as 8% in males aged > 60 years and females aged >65 years.^2^ There is no effective pharmacological treatment to prevent, delay or reverse AAA^3^ so a novel pharmacotherapy is urgently needed. The lack of effective drug therapy for AAA is partially due to a poor understanding of the molecular mechanisms that underlie its development. It has been well documented that senescence of vascular smooth muscle cells (VSMCs), the principal resident cells of the aortic wall, plays a critical role in the formation and progression of AAA.^4^ Senescent VSMCs release a variety of pro‐inflammatory cytokines and matrix‐degrading molecules such as monocyte chemotactic protein‐1, interleukin‐6 and matrix metalloproteinase 2, that contribute to AAA formation.^5^ Immunoglobulin E has been shown to activate the lincRNAp21‐p21 signalling pathway to induce VSMC senescence and thus facilitate Angiotensin II (Ang II)‐induced AAA formation in ApoE^‐/‐^ mice.^6^ Nevertheless, the precise mechanism underlying VSMC senescence in AAA is not well understood.

MicroRNAs (miRNAs) are small non‐coding RNAs (~21‐23 nucleotides) that bind to the 3’untranslated region (UTR) of specific target mRNA, inducing target degradation or inhibiting translation.^7^ miRNAs are involved in a wide range of biological and pathophysiological processes within the vasculature.^8^ Recently, it has been well demonstrated that miRNAs participate in the initiation and progression of AAA.^9^ miR‐712 has been shown to promote AAA development in Ang II–infused ApoE^‐/‐^ mice by repressing two matrix metalloproteinase inhibitors: TIMP3 and RECK.^10^ miR‐155‐5p suppresses the viability of VSMCs by targeting FOS and ZIC3 to trigger progression of AAA.^11^ Nonetheless whether and how miRNAs affect VSMC senescence in AAA remains unclear.

Sirtuins, a family of nicotinamide adenine dinucleotide‐dependent enzymes, are widely expressed in mammals. Seven sirtuins (Sirt1‐7) have been reported in humans and serve multiple functions including cell proliferation, survival and homeostasis.^12^ Sirt1, one of the best‐studied sirtuins, plays an essential role in mediating both replicative and premature cellular senescence.^13^ Previous studies revealed that Sirt1 is highly expressed in the vasculature, including by VSMCs, endothelial progenitor cells and endothelial cells, regulating cardiovascular functions.^14^, ^15^, ^16^ Indeed, an aberrant Sirt1 level is closely associated with AAA formation and progression.^17^, ^18^ Despite this, whether specific miRNA affects VSMC senescence by regulating Sirt1 to regulate AAA formation remains to be determined. This study revealed that expression of miR‐199a‐5p was significantly increased in the plasma of AAA patients and Ang II–treated VSMCs and contributed to Ang II–induced VSMC senescence by targeting Sirt1. This may provide an alternative strategy for AAA disease.

## 2 MATERIALS AND METHODS

### 2.1 Isolation, culture and characterization of VSMCs

Abdominal aortic aneurysm tissue was collected from patients who underwent surgical repair. Healthy human abdominal aortic tissue was harvested from donors and served as the control group. Written informed consent was obtained from all study patients. All procedures that involved human samples were approved by the research ethics board of Guangdong Provincial People's Hospital (No. GDREC2018060H). VSMCs were isolated from the abdominal aortic tissue as described in our previous study.^4^ Briefly, after cleaning the fatty tissue, the medial tissue of aortic tissues was carefully dissected from the adventitia and intima and then cut into 1‐2 mm^3^ pieces. Pieces were then transferred to 10‐cm poly‐L‐lysine‐coated culture plates and incubated for adhesion at 37°C for 1 hour. After attaching to the plate, the medial pieces were gently cultured with Dulbecco's modified Eagle medium (DMEM; Sigma‐Aldrich) containing 10% foetal bovine serum (FBS; Gibco) and 100 µg/mL penicillin and streptomycin (P/S, Thermo Fisher Scientific). Cell cultures were maintained at 37°C in a humidified 5% CO_2_ atmosphere. The medial pieces were left undisturbed to prevent detachment for 4 days, and the medium refreshed approximately every 3 to 4 days. VSMCs migrated out from the medial pieces within 1‐2 weeks. After removing the medial pieces, VSMCs were regularly collected and passaged. All VSMCs at passage 2−3 were used in this study. In the current study, we collected seven control‐VSMC cell lines from healthy donors (51.71 ± 3.30 years old) and eight AAA‐VSMC cell lines from AAA patients (55.25 ± 4.861 years old).

### 2.2 HE staining

Abdominal aortic tissue from AAA patients and healthy aortic tissue from control donors were harvested. After fixation with 10% formalin, the tissue was embedded in paraffin and cut into 5‐μm‐thick sections. Sections were stained with haematoxylin and eosin (HE) according to the protocol of our lab. Briefly, the sections were deparaffinized in xylene and then dehydrated in alcohol. Subsequently, the sections were stained with haematoxylin solution for 5 minutes and then rinsed in alcohol. The sections were then counterstained in the eosin solution for 30 seconds, then mounted and photographed.

### 2.3 DHE staining

The reactive oxygen species (ROS) production in AAA tissue and control aneurysmal tissue was evaluated by dihydroethidium (DHE) staining (Thermo Fisher Scientific, D1168). Briefly, sections were hydrated and incubated at room temperature with 10 μM DHE in the dark for half an hour. After washing with PBS, randomly selected areas were photographed using a fluorescence microscope and fluorescence intensity calculated using Image J software.

### 2.4 Immunofluorescent staining

VSMCs were cultured on cover slides in 24‐well plates. After washing with PBS three times, they were fixed in 4% PFA for 15 minutes followed by permeabilization with 0.1% Triton X‐100 in PBS for half an hour. Cells were blocked by 10% BSA and then incubated with the following primary antibodies at 4°C overnight: anti‐calponin (1:100, Abcam, ab46794), anti‐α‐SMA (1:100, Abcam, ab5694), anti‐MYH11 (1:100, Abcam, ab82541), anti‐Smoothelin (1:100, Abcam, ab8969), p‐p53 (1:100, Abcam, ab33889) and anti‐ki‐67 (1:100, Abcam, ab15580). Next, cells were incubated in the dark with fluorescent‐labelled secondary antibodies (1:1000) for 1 hour at room temperature. Subsequently, VSMCs were washed with PBS three times and mounted with 4′, 6‐diamidino‐2‐phenylindole (DAPI). Finally, five randomly selected areas of each slide were photographed under a fluorescence microscope.

### 2.5 Senescence‐associated β‐galactosidase (SA‐β‐gal) assay

The senescence of VSMCs was determined using a SA‐β‐gal assay kit according to the manufacturer's protocol (Beyotime, C0602). Control‐VSMCs and AAA‐VSMCs were cultured on 6‐well plates. Some control‐VSMCs were treated for 48 hour with 20 nM Ang II or simultaneously combined with 10 nM NAC (Santa Cruz, SC‐221945) or 100 nM Resveratrol (Sigma‐Aldrich, R5010). After washing three times with PBS, cells were fixed for 30 minutes, then stained with SA‐β‐gal solution at 4°C overnight (without CO_2_). Finally, cells were washed with PBS and randomly photographed. The percentage of SA‐β‐gal positive cells was analysed from five different view fields of each sample in three independent experiments. The percentage of SA‐β‐gal positive cells was calculated to estimate the percentage of senescent cells.

### 2.6 Bromodeoxyuridine (Brdu) incorporation assay

The proliferation of VSMCs was evaluated using a BrdU incorporation kit according to the protocol (Roche, 11647229001**)**. Briefly, 3 × 10^4^ VSMCs were seeded in 96‐well plates and incubated at 37℃with 10 μM BrdU labelling solution for 24 hours. After removing labelling solution, the VSMCs were treated with 200 μL FixDenat solution for 30 minutes. Next, the cells were incubated with anti‐BrdU‐POD working solution for 90 minutes. Finally, after washing with PBS three times, the cells were incubated with 100 μL substrate solution for 5 minutes and the absorbance at 450 nm measured.

### 2.7 Transfection with miR‐199a‐5p mimics, miR‐199a‐5p inhibitors or miR‐control

The miR‐control, miR‐199a‐5p mimics and miR‐199a‐5p inhibitors were purchased from GenePharma. VSMCs were transfected with miR‐199a‐5p mimics, miR‐199a‐5p inhibitors or miR‐control (50nM) using Lipofectamine 2000 (11668027, Invitrogen) according to the manufacturer's instructions.

### 2.8 H_2_DCFDA staining

To detect the generation of ROS in VSMCs, H_2_DCFDA staining (D399, Invitrogen) was performed according to the protocol. Briefly, control‐VSMCs were cultured in 24‐well plates with collagen‐coated glass coverslips, then treated with Ang II or Ang II + NAC. Cells were then incubated in the dark with 10 μM H_2_DCFDA for 15 minutes at 37°C. Five different view fields of each sample were photographed and fluorescence intensity calculated in three independent experiments using Image J software.

### 2.9 Real‐time PCR

Total RNA from VSMCs or serum was isolated with TRIzol reagent (Takara, RNAiso Plus, 9108 and RNAiso Blood, 9112). Reverse transcription was performed using a PrimeScript RT Reagent Kit (Takara, RR037A), and qRT‐PCR of miR‐199a‐5p performed using a One‐Step TB Green^®^ PrimeScript^™^ RT‐PCR Kit (Takara, RR820A). For miR‐199a‐5p, Bulge‐Loop^™^ miRNA RT primer (RiboBio) was used. U6 was the reference gene for miRNA expression analysis. The expression of miR‐199a‐5p was normalized to the expression of U6 using the 2^−ΔΔCt^ cycle threshold method. The experiments were repeated at least three times.

### 2.10 Western blotting

Total protein of treated VSMCs was extracted using RIPA (CST, 9806) with Protease/Phosphatase inhibitor (CST, 5872) and the concentration measured using a bicinchoninic acid (BCA) assay kit (Thermo, 231227). A total of 30 µg protein was resolved by 10% Tris‐glycine gel electrophoresis and then transferred onto a PVDF membrane. After blocking with 5% fat‐free milk in TBST, the PVDF membranes were incubated overnight at 4°C with the following antibodies: anti‐p53 (Abcam, ab26), anti‐p‐p53 (Abcam, ab33889), anti‐p21 (Abcam, ab109199), anti‐Sirt1 (Abcam, ab110304) and anti‐GAPDH (CST, 2118). Membranes were then washed three times with TBST and incubated with secondary antibodies (1:3000, CST) at room temperature for 1 hour and then exposed in a dark room. The quantification of Western blotting in three independent experiments was analysed using Image J software (National Institutes of Health).

### 2.11 Cumulative population doubling

Total number of VSMCs at the same passage in different groups were counted and cells (6 × 10^3^) plated onto 96‐well dishes. After reaching 90% confluence, they were trypsinized, counted and reseeded. Cumulative population doubling (CDP) of VSMCs was calculated with the following formulae: Xp = [log(10)Np ‐ log(10)Np ‐ 1] / log(10)2(Np, final number of cells; Np‐1, initial number of cells; X: population doubling), CDP = Xp + Xp ‐ 1. The final passage of long‐term culture was the passage in which cells did not proliferate twice or lost their fibroblastic shape.

### 2.12 microRNA sequencing and data analysis

Total RNA was extracted from the serum of patients and purified using a miRNeasy^®^ Mini kit (Qiagen, 217004) according to the manufacturer's protocol. Subsequently, the degradation and contamination of RNA were assessed and the concentration and purity of RNA measured. After ligating into 18‐30 nt, small RNAs were reverse‐transcribed to cDNA and a cDNA library generated. Gene Denovo Biotechnology Co. sequenced cDNA using Illumina HiSeqTM 2500. Raw reads were further filtered, then microRNA aligned and identified. miRNA expression profiles, miRNA principal component, miRNA Expression Pattern Clustering, differentially expressed miRNA (DE miRNA), Target gene Prediction and Target gene functional enrichment were analysed. The heatmaps of miRNA related to Sirt1 were drawn to display miRNA expression level in different patients and to cluster miRNAs with a similar expression pattern. We identified miRNAs with a fold change ≥2 and *P*‐value < .05 in a comparison as significant DE miRNAs.

### 2.13 Luciferase activity assay

Wild‐type SIRT1 3′UTR firefly luciferase reporter plasmids and SIRT1 3′UTR firefly luciferase reporter plasmids with the potential miR‐199a‐5p‐binding site mutated were used in this study. These plasmids were co‐transfected, respectively, with miR‐199a‐5p mimic or miR‐control to HEK293 cells, while renilla luciferase reporter plasmids were also transfected and served as an internal control. After transfection, luciferase activity was detected using a Dual‐Glo Luciferase Assay Kit (Promega).

### 2.14 Viral vector construction and infection

The lentiviral plasmid constructs for Sirt1 in VSMCs were purchased from TranSheepBio (J0510‐A9, TranSheepBio). The lentivirus was packaged as previously described.^19^ VSMCs at a confluence of 70‐80% were infected by lentivirus at a multiplicity of infection of 10 with polybrene (8 μg/mL). Transfection efficiency was determined after 72 hours by Western blotting.

### 2.15 ELISA assay

The concentration of IL‐6 and TNF‐α in aortic tissue was measured using a human IL‐6 Quantikine^®^ ELISA Kit (R&D Systems, D6050) and human TNF‐α Quantikine^®^ ELISA Kit (R&D Systems, STA00D) according to the manufacturer's instructions.

### 2.16 Statistical analysis

All values are expressed as mean ± SEM. Analysis was performed using GraphPad Prism Software. Statistical significance was determined by independent‐samples T test between two groups or analysis of variance (ANOVA) followed by Bonferroni test between more than two groups. *P <* .05 was considered statistically significant.

## 3 RESULTS

### 3.1 VSMCs exhibit cellular senescence in human AAA tissue

**FIGURE 1:**
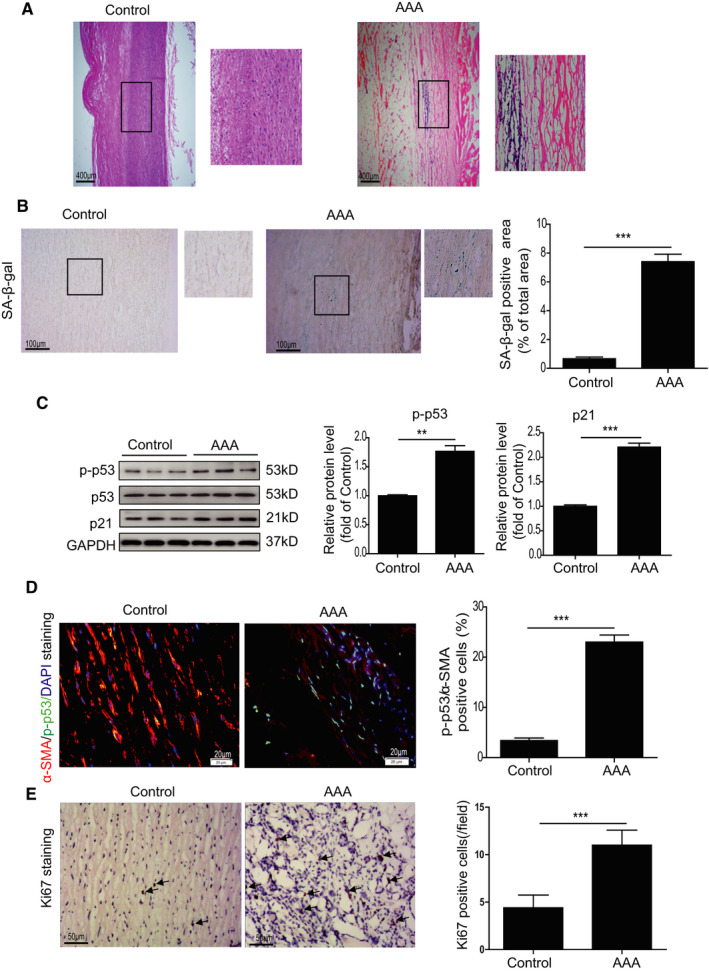
VSMCs displayed cellular senescence in human AAA tissues. A, Representative images of HE‐stained sections of abdominal aorta from control donors and AAA patients. HE staining revealed vasculopathy in the medial layer of the abdominal aorta in AAA patients. B, Representative images and quantitative analysis of SA‐β‐gal assay in the abdominal aorta from control donors and AAA patients. C, Western blotting and quantitative analysis of p‐p53 and p21 in the abdominal aorta of control donors and AAA patients. D, Representative images and quantitative analysis of p‐p53 (green) and α‐SMA (Red) staining in the abdominal aorta from control donors and AAA patients. E, Representative images and quantitative analysis of Ki67 staining in the abdominal aorta from control donors and AAA patients. **P < .01, ***P < .001

Our previous study showed increased VSMC senescence in aortic aneurysmal tissue from MFS patients.^4^ We, therefore, examined cellular senescence in human AAA tissue. First, HE staining revealed the vasculopathy in the medial layer of the abdominal aorta in AAA patients (Figure 1A). We next performed SA‐β‐gal assay to detect cellular senescence in AAA tissue. As shown in Figure 1B, SA‐β‐gal activity was significantly increased in AAA tissue relative to control tissue (Figure 1B). The SA‐β‐gal stained area was mainly located in the medial layer of the abdominal aorta in AAA patients, suggesting a role of medial VSMC senescence (Figure 1B). Western blotting also showed that the level of cellular senescence markers p‐p53 and p21 was much higher in AAA tissue compared with control tissue (Figure 1C). To further verify whether VSMCs are senescent in AAA tissue, we performed VSMC marker α‐SMA and p‐p53 double staining. The percentage of α‐SMA^+^ (red colour)/p‐p53^+^ (green colour) double‐positive cells (the number of α‐SMA^+^/p‐p53^+^ double‐positive cells/all DAPI (blue colour) positive cells) had increased fivefold in tissue from AAA patients compared with that of control donors (Figure 1D). Despite increased VSMC senescence, immunohistological staining for the proliferation marker Ki67 showed increased positive staining in tissue from AAA patients compared with that of control donors, suggesting a compensatory proliferative response during AAA formation (Figure 1E). These data suggest that VSMCs in AAA tissue were senescent.

### 3.2 VSMCs derived from AAA patients demonstrate cellular senescence

**FIGURE 2:**
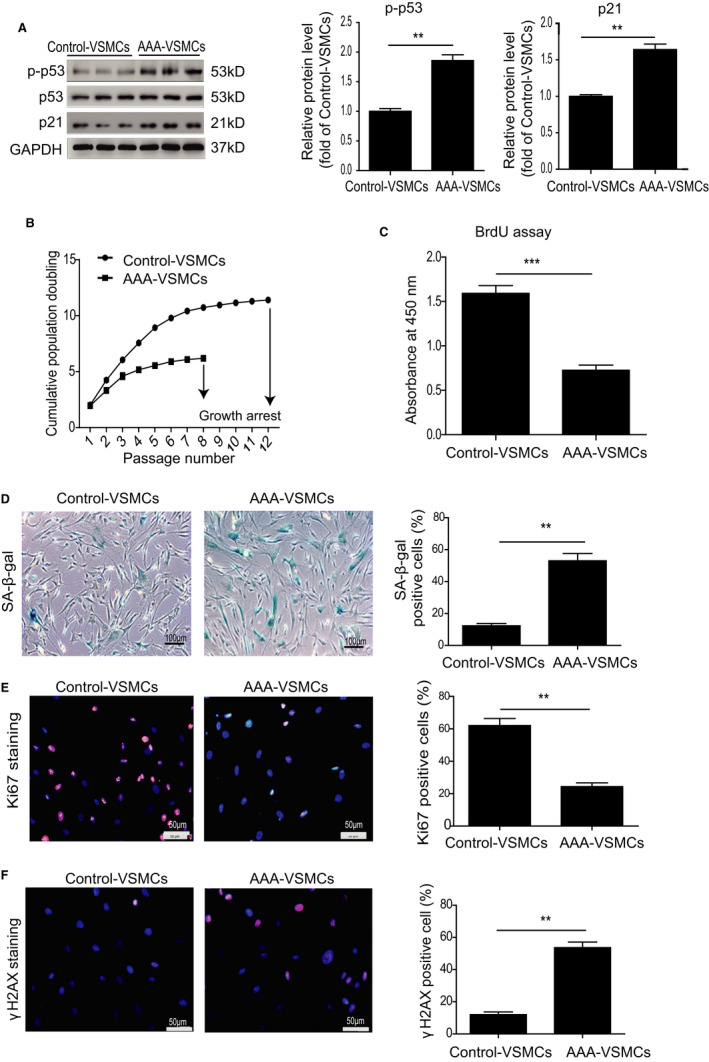
VSMCs isolated from human AAA tissue exhibited cellular senescence. A, Western blotting and quantitative analysis of the expression of p‐p53 and p21 in AAA‐VSMCs and control‐VSMCs. B, Cell growth curves display a lower growth rate of AAA‐VSMCs than control‐VSMCs. C, BrdU assay shows a lower absorbance at 450 nm of AAA‐VSMCs than control‐VSMCs. D, Representative images of SA‐β‐gal assay and quantitative analysis of SA‐β‐gal positive cells in AAA‐VSMCs and control‐VSMCs. E, Representative images of Ki67 staining and quantitative analysis of Ki67 positive cells in AAA‐VSMCs and control‐VSMCs. F, Representative images of γH2AX staining and quantitative analysis of γH2AX positive cells in AAA‐VSMCs and control‐VSMCs. **P < .01, ***P < .001

We successfully isolated VSMCs as evidenced by expression of α‐SMA, Calponin, MYH11 and Smoothelin from AAA patients and control donors (Figure S1). In the current study, we totally isolated seven control‐VSMC cell lines from healthy donors and eight AAA‐VSMC cell lines from AAA patients. Consistently, AAA‐VSMCs exhibited a higher protein level of p‐p53 and p21 than control‐VSMCs (Figure 2A). Next, we compared the cell growth rate via serial passaging: AAA‐VSMCs arrested at passage 8, whereas control‐VSMCs continued growing until passage 12, suggesting that AAA‐VSMCs had a lower growth rate (Figure 2B). BrdU assay also showed that compared with control‐VSMCs, the absorbance at 450nm of AAA‐VSMCs was decreased by 50%, indicating that their proliferation rate was reduced (Figure 2C). We performed SA‐β‐gal assay to compare cellular senescence between AAA‐VSMCs and control‐VSMCs. As shown in Figure 2D, SA‐β‐gal activity was greatly enhanced in AAA‐VSMCs compared with control‐VSMCs (Figure 2D). In contrast, the number of ki‐67 positive cells was decreased by 60% in AAA‐VSMCs compared with control‐VSMCs (Figure 2E). We also examined DNA damage in AAA‐VSMCs and control‐VSMCs using γH2AX staining. Compared with control‐VSMCs, the percentage of γH2AX positive cells had tripled in AAA‐VSMCs (Figure 2F). Collectively, these results indicate that VSMCs isolated from AAA patients are senescent.

### 3.3 Ang II induces VSMC senescence via upregulation of ROS generation

**FIGURE 3:**
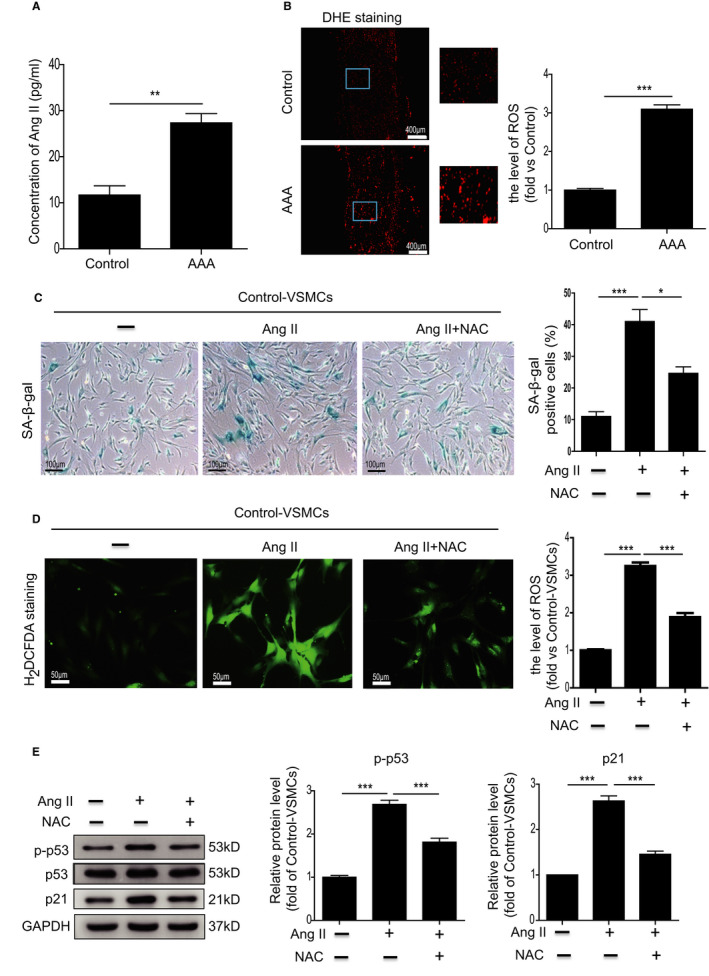
Ang II–induced cellular senescence in VSMCs via ROS generation. A, The concentration of Ang II in the serum from control donors and AAA patients was measured by ELISA. n = 7‐8. B, Representative images of DHE staining and quantitative analysis of ROS generation in the abdominal aorta from control donors and AAA patients. n = 7‐8. C, Representative images and quantitative analysis of SA‐β‐gal assay in control‐VSMCs treated with Ang II or Ang II combined with NAC. D, Representative images of H_2_DCFDA staining and quantitative analysis of ROS generation in control‐VSMCs treated with Ang II or Ang II combined with NAC. E, Western blotting and quantitative analysis of the expression of p–p53 and p21 in control‐VSMCs treated with Ang II or Ang II combined with NAC. *P < .05, **P < .01, ***P < .001

To the best of our knowledge, a high level of Ang II is a major cause of AAA formation.^20^, ^21^ We measured the level of Ang II in the plasma of AAA patients. It has been documented that the circulating Ang II level is significantly increased in acute aortic dissection patients compared with healthy controls.^22^ Consistently, compared with control donors, the concentration of Ang II was increased in AAA patients (27.3 ± 3.5 pg/mL vs. 11.6 ± 3.5 pg/mL, Figure 3A). To examine whether AngII induces VSMC senescence, we treated VSMCs with 1 nM, 10 nM, 20 nM and 50 nM AngII for 48 hours. As shown in Figure S2A, Ang II induced VSMC senescence at 20 nM but this plateaued at 50nM, indicating that the effect was dose‐dependent (Figure S2A). Next, we treated VSMCs with 20 nM AngII for 24, 48, 72 and 168 hours. AngII induced VSMC senescence at 48 hours but the effect plateaued at 72 and 168 hours (Figure S2B), suggesting that Ang II–induced VSMC senescence was dose‐ and time‐dependent. Based on these results, 20nM of AngII‐treated VSMCs for 48 hours was chosen for further studies. Since ROS generation contributes to cellular senescence, we determined ROS level in AAA tissue by DHE staining. As shown in Figure 3B, the level of ROS had tripled in AAA tissue (Figure 3B). Furthermore, ROS generation was significantly increased as evidenced by H_2_DCFDA staining in AAA‐VSMCs compared with control‐VSMCs (Figure S3A). Flow cytometry analysis also showed a higher level of intracellular ROS in AAA‐VSMCs than control‐VSMCs (Figure S3B). We then analysed whether Ang II regulates VSMC senescence via ROS production. Ang II treatment significantly enhanced the percentage of SA‐β‐gal‐positive cells among VSMCs and increased ROS generation (Figure 3C,D), whereas ROS scavenger NAC remarkably inhibited VSMC senescence and ROS generation. Moreover, NAC inhibited the upregulation of p‐p53 and p21 expression induced by Ang II (Figure 3E). The above results suggest that Ang II mediates VSMC senescence via ROS production.

### 3.4 Ang II induces VSMC senescence though regulation of Sirt1

**FIGURE 4:**
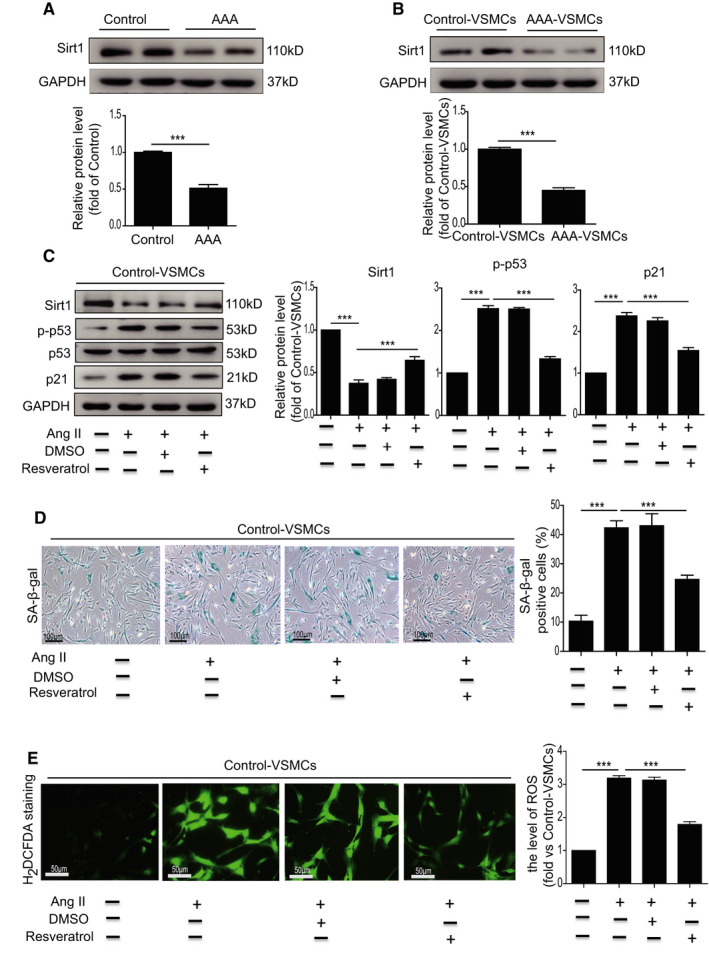
Ang II–induced VSMC senescence though regulation of Sirt1. A, Western blotting and quantitative analysis of the expression of p–p53 and p21 in the abdominal aorta from control donors and AAA patients. B, Western blotting and quantitative analysis of the expression of p–p53 and p21 in AAA‐VSMCs and control‐VSMCs. C, Western blotting and quantitative analysis of the expression of Sirt1, p–p53 and p21 in control‐VSMCs treated with Ang II or Ang II combined with DMSO or resveratrol. D, Representative images and quantitative analysis of SA‐β‐gal assay in control‐VSMCs treated with Ang II or Ang II combined with DMSO or resveratrol. E, Representative images of H_2_DCFDA staining and quantitative analysis of ROS generation in control‐VSMCs treated with Ang II or Ang II combined with DMSO or resveratrol. ***P < .001

Accumulating evidence has shown that Sirt1 plays a critical role in regulating ROS generation^23^, ^24^ with a complex interplay between the two. Increased ROS can directly or indirectly regulate Sirt1 activity, which in turn controls ROS level.^25^, ^26^ The exact relationship between Sirt1 activity and ROS generation may depend on the cell types and the cellular contexts. To establish the relationship between VSMC senescence and Sirt1 and ROS in the current study, we first detected the expression of Sirt1 in human AAA tissue. The level of Sirt1 in human AAA tissue was significantly lower than in control tissues (Figure 4A), and likewise significantly reduced in AAA‐VSMCs compared with control‐VSMCs (Figure 4B). We then investigated the involvement of Sirt1 in Ang II–induced VSMC senescence and established that Sirt1 was significantly decreased (Figure 4C) in Ang II–treated VSMCs, accompanied by a significant increase in protein level of p‐p53 and p21 (Figure 4C) as well as cellular senescence (Figure 4D) and ROS production (Figure 4E). Nevertheless Resveratrol, a Sirt1 activator, significantly downregulated the increased level of p‐p53 and p21 as well as cellular senescence and ROS production in Ang II–treated VSMCs (Figure 4C‐E). To further verify the role of Sirt1 in Ang II–induced VSMC senescence, we overexpressed Sirt1 in control‐VSMCs and then treated the cells with Ang II. As shown in Figure S4, overexpressed Sirt1 rescued Ang II–induced VSMC senescence (Figure S4A). Moreover, overexpressed Sirt1 inhibited Ang II–induced ROS generation in VSMCs (Figure S4B).

In addition to ROS, downregulation of Sirt1 is closely associated with inflammation that contributes to AAA formation.^27^ We examined the expression of IL‐6 and TNF‐α in AAA tissue. Compared with control tissue, the concentration of IL‐6 and TNF‐α was significantly increased in AAA tissue compared with control tissue (Figure S5). These results indicate that Ang II induces VSMC senescence though downregulation of Sirt1.

### 3.5 miR‐199a‐5p mediates Sirt1 expression

**FIGURE 5:**
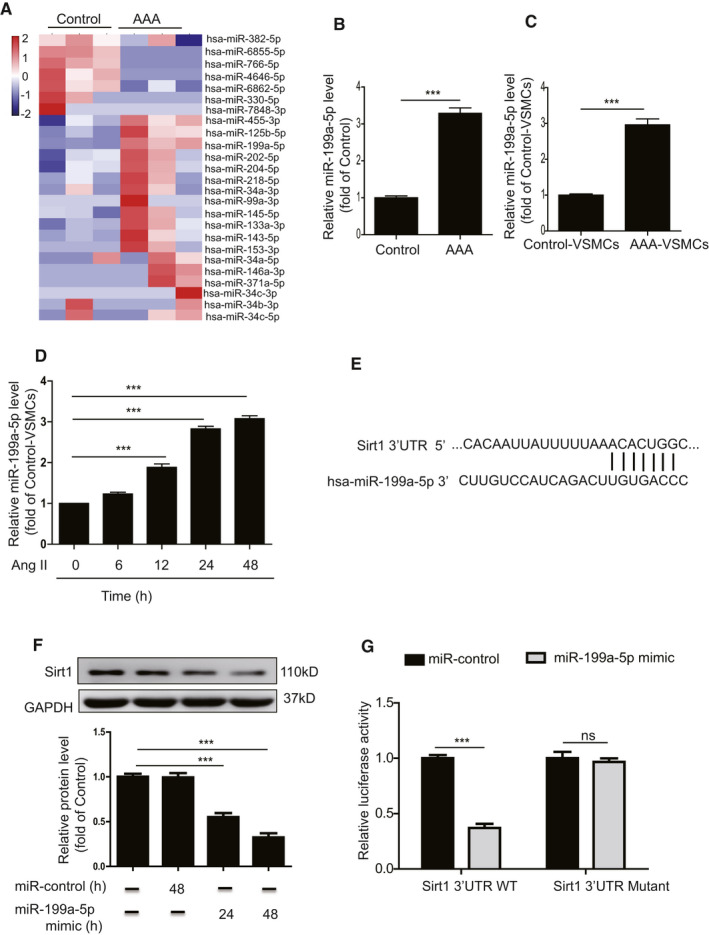
miR‐199a‐5p mediates Sirt1 expression. A, Heat map showing upregulated and downregulated miRNAs in plasma isolated from control donors and AAA patients. B, miR‐199a‐5p level in the abdominal aorta from control donors and AAA patients was examined by qRT‐PCR. C, miR‐199a‐5p level in AAA‐VSMCs and control‐VSMCs was examined by qRT‐PCR. D, miR‐199a‐5p level 6, 12, 24 and 48 hours after Ang II–treated VSMCs were analysed by qRT‐PCR. E, Analysis of Sirt1 3′‐UTR binding site for miR‐199a‐5p. F, Western blotting and quantitative analysis of the expression of Sirt1 in control‐VMSCs treated with miR‐control or miR‐199a‐5p mimic. G, miR‐199a‐5p inhibited the luciferase activity of Sirt1 with wild‐type 3′‐UTR but had no impact on that of Sirt1 with mutant‐type 3′‐UTR. ***P < .001. ns: not significant

Increasing evidence has shown that miRNAs play a vital role in regulating AAA formation. To investigate whether miRNA(s) regulate the expression of Sirt1 to mediate VSMC senescence, we examined the expression of miRNAs collected from the plasma of AAA patients and control donors using miRNA‐sequencing. The results revealed that AAA patients had a significantly different miRNA expression signature to control donors (Figure 5A). The miR‐199a‐5p level was significantly upregulated in AAA patients (Figure 5B) and also greatly enhanced in AAA‐VSMCs compared with control‐VSMCs (Figure 5C). Furthermore, Ang II treatment upregulated the level of miR‐199a‐5p in VSMCs in a time‐dependent manner (Figure 5D). Bioinformatic analysis using TargetScan (http://www.targetscan.org/) showed that the 3′‐UTR of Sirt1 has a potential‐binding site for miR‐199a‐5p (Figure 5E), suggesting that Sirt1 is a potential target of miR‐199a‐5p. Next, we examined whether overexpression of miR‐199a‐5p could alter the expression of Sirt1 in VSMCs. The results showed that miR‐199a‐5p mimic treatment significantly downregulated the expression of Sirt1 in VSMCs (Figure 5F). Furthermore, a dual‐luciferase reporter gene assay demonstrated that the miR‐199a‐5p mimic significantly reduced the luciferase activity of the Sirt1 wild‐type (WT) reporter but had no influence on that of the Sirt1 mutant reporter (Figure 5G). In summary, these results indicate that miR‐199a‐5p regulates Sirt1 expression.

### 3.6 Ang II induces VSMC senescence through targeting of Sirt1 by miR‐199a‐5p

**FIGURE 6:**
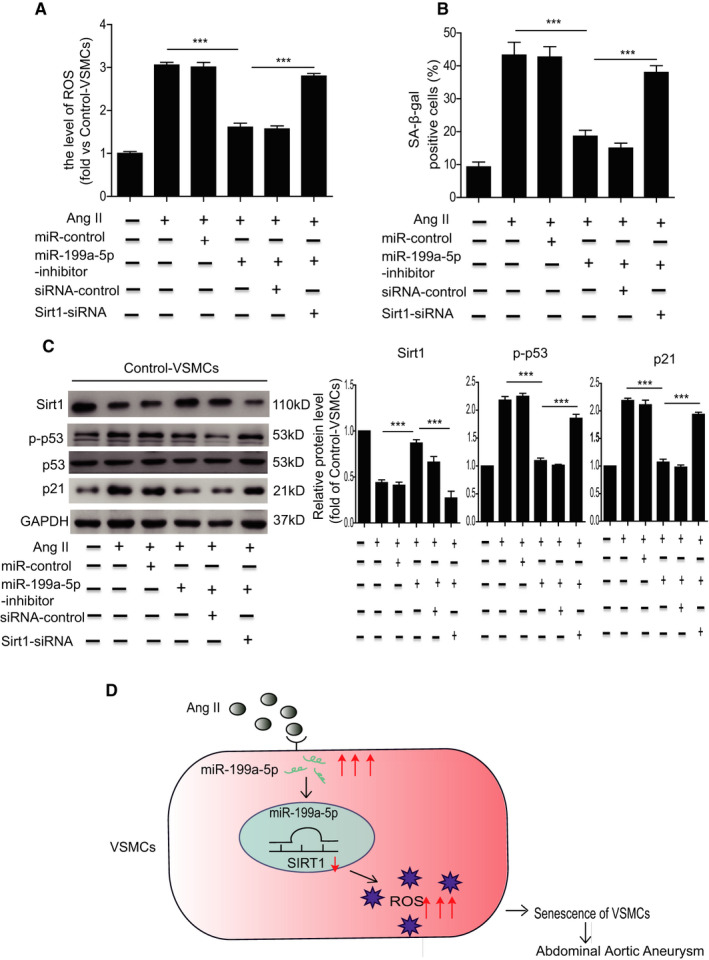
Ang II–induced VSMC senescence through miR‐199a‐5p targeting Sirt1. A, Quantitative analysis of ROS generation in control‐VSMCs treated with Ang II, Ang II + miR‐control, Ang II + miR‐199a‐5p inhibitor, Ang II + miR‐199a‐5p inhibitor + siRNA‐control and Ang II + miR‐199a‐5p inhibitor + Sirt1‐siRNA. B, Quantitative analysis of SA‐β‐gal positive cells in control‐VSMCs treated with Ang II, Ang II + miR‐control, Ang II + miR‐199a‐5p inhibitor, Ang II + miR‐199a‐5p inhibitor + siRNA‐control and Ang II + miR‐199a‐5p inhibitor + Sirt1‐siRNA. C, Western blotting and quantitative analysis of the expression of Sirt1, p‐p53 and p21 in control‐VSMCs treated with Ang II, Ang II + miR‐control, Ang II + miR‐199a‐5p inhibitor, Ang II + miR‐199a‐5p inhibitor + siRNA‐control and Ang II + miR‐199a‐5p inhibitor + Sirt1‐siRNA. D, Proposed mechanisms for miR‐199a‐5p regulation of VSMC senescence in AAA. ***P < .001

We verified the functional role of miR‐199a‐5p in the regulation of Sirt1‐mediated Ang II–induced VSMC senescence. Inhibition of miR‐199a‐5p using miR‐199a‐5p inhibitor greatly attenuated ROS generation (Figure 6A) and cellular senescence (Figure 6B) induced by Ang II in VSMCs. Inhibition of miR‐199a‐5p upregulated the expression of Sirt 1 and downregulated the expression of p‐p53 and p21 (Figure 6C) in Ang II–treated VSMCs. To confirm that Sirt1 is a target of miR‐199a‐5p, VSMCs were co‐transfected with miR‐199a‐5p inhibitor and Sirt1‐siRNA. The results showed that Sirt1‐siRNA partially abrogated the effect of miR‐199a‐5p inhibition on ROS generation, cellular senescence and Sirt1, and p‐p53/p21 expression in Ang II–treated VSMCs (Figure 6A‐C). These data demonstrate that miR‐199a‐5p/Sirt1 signalling is involved in Ang II–induced VSMC senescence.

## 4 DISCUSSION

There are several major findings in the current study (Figure 6D). First, VSMCs in patients with AAA exhibited cellular senescence. Second, Sirt1 expression was downregulated in human AAA tissue. Elevation of the Ang II level caused VSMC senescence by downregulation of Sirt1 in vitro. Third, miR‐199a‐5p aggravated Ang II–induced VSMC senescence via targeting of Sirt1, and inhibition of miR‐199a‐5p attenuated Ang II–induced VSMC senescence. Our study showed that miR‐199a‐5p/Sirt1 signalling plays a critical role in Ang II–induced VSMC senescence.

Despite advanced developments in conventional therapy for AAA, the molecular mechanisms underlying AAA formation and progression are not fully understood. VSMCs, which are the major cell type in the media layer of blood vessels, maintain vessel architecture and remodelling, thereby regulating blood pressure and flow. VSMCs exhibit contractile (differentiated) and synthetic (dedifferentiated) phenotypes under different conditions, and is essential to maintain their function.^28^ There is increasing recognition that VSMC injury causes phenotypic transition with consequent vessel wall dysfunction and formation of AAA.^29^, ^30^ VSMC senescence plays a critical role in regulation of this phenotypic transition.^31^ It has been reported that milk fat globule‐epidermal growth factor 8 promotes the proinflammatory phenotypic shift of VSMCs via activation of the NF‐κB signalling pathway, leading to VSMC senescence that contributes to arterial disease.^32^ In Ang II–treated mice, exposure to particulate matter 2.5 has been shown to induce aortic smooth muscle cell senescence as manifested by increased expression of p21, p16 and SA‐β‐gal activity, therefore leading to an increased incidence of AAA.^33^ Since there are several types of cells, including VSMCs, endothelial cells and vascular progenitor cells, in aortic tissue, we performed SMA and p53 double staining to determine whether VSMCs were senescent in the aortic tissue of patients with AAA. The number of α‐SMA^+^/p53^+^ double‐positive cells was significantly increased in human AAA tissue compared with tissue from control donors. Furthermore, AAA‐VSMCs had a lower growth rate accompanied by increased DNA damage and SA‐β‐gal activity in culture. These results suggest that VSMCs in human AAA tissue are senescent although the underlying mechanisms remain unclear.

There is strong evidence that Sirt1 plays a critical role in the regulation of vascular aging. Sirt1 expression is inversely associated with vascular aging: its deficiency in endothelial cells, monocytes/macrophages and VSMCs promotes vascular ageing.^34^, ^35^ Age‐related loss of Sirt1 leads to VSMC dysfunction including reduced proliferation capacity and increased senescence, inducing vascular diseases.^36^ Treatment with resveratrol, a Sirt1 activator, markedly decreases the number of SA‐β‐gal‐stained cells in Ang II–treated VSMCs via elevation of Sirt1 expression.^37^ Moreover, resveratrol treatment dramatically attenuates arterial aging in aging mice. VSMC‐specific knockout of Sirt1 accelerates Ang II–induced AAA formation via induction of VSMC senescence, whereas overexpression of Sirt1 in VSMCs ameliorates Ang II–induced senescence, therefore suppressing AAA formation in Apoe^−/−^ mice.^27^ These results strongly suggest that Sirt1‐mediated VSMC senescence is closely related to vascular diseases. In this study, we also found that Sirt1 expression was greatly reduced in human AAA tissue, AAA‐VSMCs and Ang II–treated VSMCs. Furthermore, resveratrol treatment significantly inhibited Ang II–induced VSMC senescence. Several clinical trials studying the therapeutic effects of resveratrol on preventing cardiovascular disease and artery disease are underway (ClinicalTrials.gov. Unique identifier: NCT02409537, NCT03743636). Sirt1 expression should be considered in future trials of resveratrol therapies in patients with AAA. Since Sirt1 plays a critical role in SMC senescence, it would be relevant to future clinical application to determine whether miR199a‐5p inhibition or resveratrol more potently attenuates Ang II–induced SMC senescence. Although the molecular mechanisms underlying AAA remain unclear, miRNAs have emerged as key regulators of AAA formation and development.^3^ miR‐33a‐5p expression is significantly enhanced in human AAA tissue whereas miR‐33 deletion attenuates Ang II‐ and calcium chloride‐induced AAA formation in mice via upregulation of ATP‐binding cassette transporter A1 expression.^38^ miR‐181b is highly overexpressed in AAA tissue and administration of locked nucleic acid anti‐miR‐181b retards AAA development by upregulating tissue inhibitor metalloproteinase‐3 and elastin in Ang II–infused Apoe‐/‐ mice.^39^ Since Sirt1 expression was greatly reduced in AAA tissue, we presume the presence of a potential miRNA that targets Sirt1. Aging‐associated increased miR‐34a expression promotes VSMC senescence by inhibiting Sirt1, leading to arterial injury.^40^ Compared with VSMCs isolated from miR‐34a^+/+^ mice, those from miR‐34a^‐/‐^ mice are less prone to senescence.^41^ We analysed our miRNA sequencing data and found that miR‐34 was also upregulated in AAA patients compared with control donors. Nonetheless its expression was not high among those miRNAs with increased expression in AAA patients. This may be due to the different miRNA expression signatures of AAA patients and AAA mice. In addition to miR‐34a, whether other miRNAs induce VSMC senescence in AAA via targeting of Sirt1 needs to be determined. We found that miR‐199a‐5p was greatly enhanced in AAA patients, AAA‐VSMCs and Ang II–treated VSMCs. Bioinformatic analysis showed that miR‐199a‐5p can bind to Sirt1 via 3′‐UTR. In this study, we showed that Sirt1 is regulated by miR‐199a‐5p. MiR‐199a‐5p mimic treatment significantly reduced Sirt1 expression in VSMCs. Luciferase assay verified this observation. We also observed that miR‐199a‐5p inhibitor treatment significantly reduced Ang II‐induced VSMC senescence via upregulation of Sirt1 and this effect was abrogated by Sirt1‐siRNA.

There are some limitations that we should acknowledge. First, we investigated the role of only miR‐199a‐5p in regulation of VSMC senescence. Whether other miRNAs that were significantly upregulated in AAA patients including miR‐455‐3p or miR‐125b‐5p mediate VSMC senescence requires further investigation. Second, whether miR‐199a‐5p contributes to AAA formation via regulation of VSMC senescence needs to be further verified in Ang II–infused Apoe^‐/‐^ mice. Third, it has been reported that SHNG12 targeting miR‐199a‐5p/HIF‐1α contributed to atherosclerosis formation by mediating the phenotypes of VSMCs.^42^ In addition to Sirt1, whether miR‐199a‐5p mediates VSMC senescence via regulation of other targets needs to be addressed in future study. Fourth, whether miR‐199a‐5p can injure other cell types including endothelial cells and fibroblasts to promote AAA formation has not been demonstrated.

In summary, our study shows that Ang II activates the miR‐199a‐5p/Sirt1 pathway to induce VSMC senescence and this contributes to AAA formation. This study explored a novel molecular mechanism of AAA formation and provides a new therapeutic strategy for AAA.

## CONFLICT OF INTERESTS

The authors declare that there are no competing interests associated with the manuscript.

## AUTHOR CONTRIBUTION

**Wuyuan Tao:** Conceptualization (supporting); Data curation (supporting); Investigation (supporting); Methodology (supporting); Writing‐original draft (supporting). **Yimei Hong:** Data curation (supporting); Methodology (supporting); Validation (supporting). **Haiwei He:** Data curation (supporting); Formal analysis (supporting); Investigation (supporting). **Qian Han:** Data curation (supporting); Investigation (supporting); Methodology (supporting); Resources (supporting). **Mengmeng Mao:** Data curation (supporting); Formal analysis (supporting); Methodology (supporting). **Bei Hu:** Data curation (supporting); Formal analysis (supporting); Methodology (supporting); Resources (supporting). **Hao Zhang:** Data curation (supporting); Methodology (supporting). **Xiaoran Huang:** Data curation (supporting); Methodology (supporting). **Wei You:** Data curation (supporting); Formal analysis (supporting); Investigation (supporting); Methodology (supporting). **Xiaoting Liang:** Data curation (supporting); Formal analysis (supporting); Methodology (supporting). **Yuelin Zhang:** Conceptualization (lead); Data curation (lead); Funding acquisition (lead); Project administration (lead); Supervision (lead); Writing‐original draft (lead); Writing‐review & editing (lead). **Xin Li:** Conceptualization (lead); Project administration (lead); Resources (lead); Writing‐original draft (lead); Writing‐review & editing (lead).

## Supporting information

Fig S1‐S5

## Data Availability

The data used to support the findings of this study are available from the corresponding author on reasonable request.
