# Supplementary material for: MicroRNA‐199a‐5p aggravates angiotensin II–induced vascular smooth muscle cell senescence by targeting Sirtuin‐1 in abdominal aortic aneurysm
Source: J Cell Mol Med. 2021 Jun 15;25(13):6056–69. doi: 10.1111/jcmm.16485 (PMC8366448; doi:10.1111/jcmm.16485)

**Supplemental information**

**Supplemental Figure 1. VSMCs were isolated from abdominal aorta from control donors and AAA patients.**

Representative images of immunofluorescent staining for α-SMA, Calponin, MYH11 and Smoothelin in control-VSMCs and AAA-VSMCs.


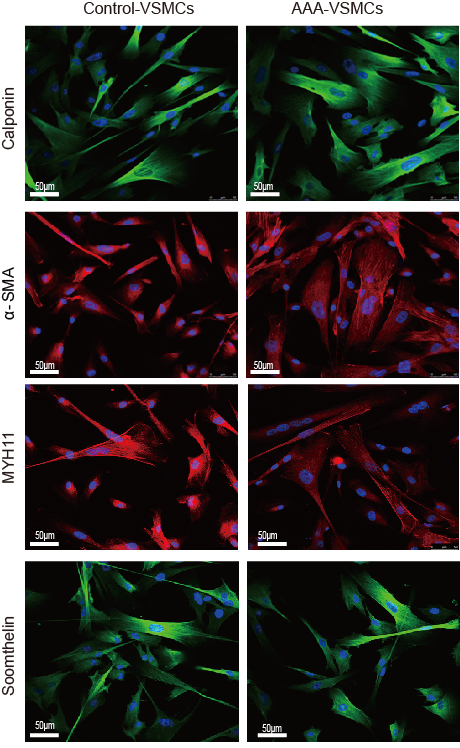


**Supplemental Figure 2. Ang II induced VSMC senescence in a dose and time-dependent manner.**

(A) Representative images and quantitative analysis of SA-β-gal staining in control-VSMCs treated with 1, 10, 20 and 50 nM Ang II for 48 hours. (B) Quantitative analysis of SA-β-gal staining in control-VSMCs treated with 20 nM Ang II for 24, 48, 72 and 168 hours, respectively. ****p<0.001*. ns=not significant.


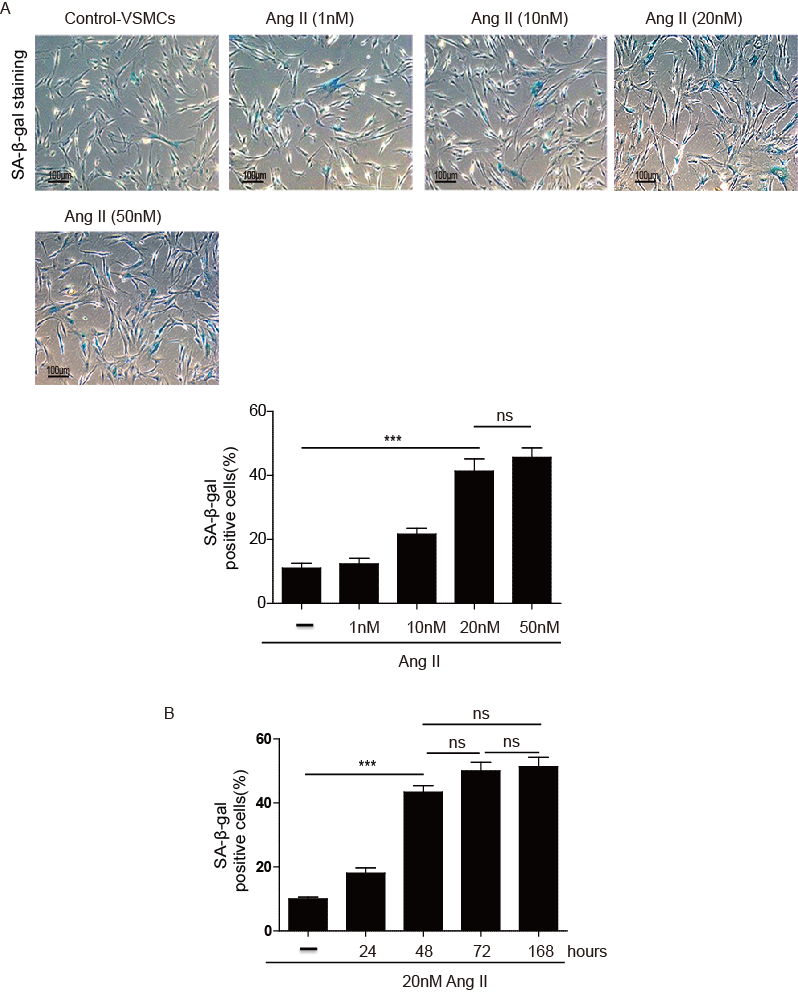


**Supplemental Figure 3. ROS generation was measured in control-VSMCs and AAA-VSMCs.**

(A) Representative images of H_2_DCFDA staining and quantitative analysis of ROS generation in control-VSMCs and AAA-VSMCs. (B) Intracellular ROS level was measured with H_2_DCFDA staining by flow cytometry analysis in control-VSMCs and AAA-VSMCs. ***p<0.001.*


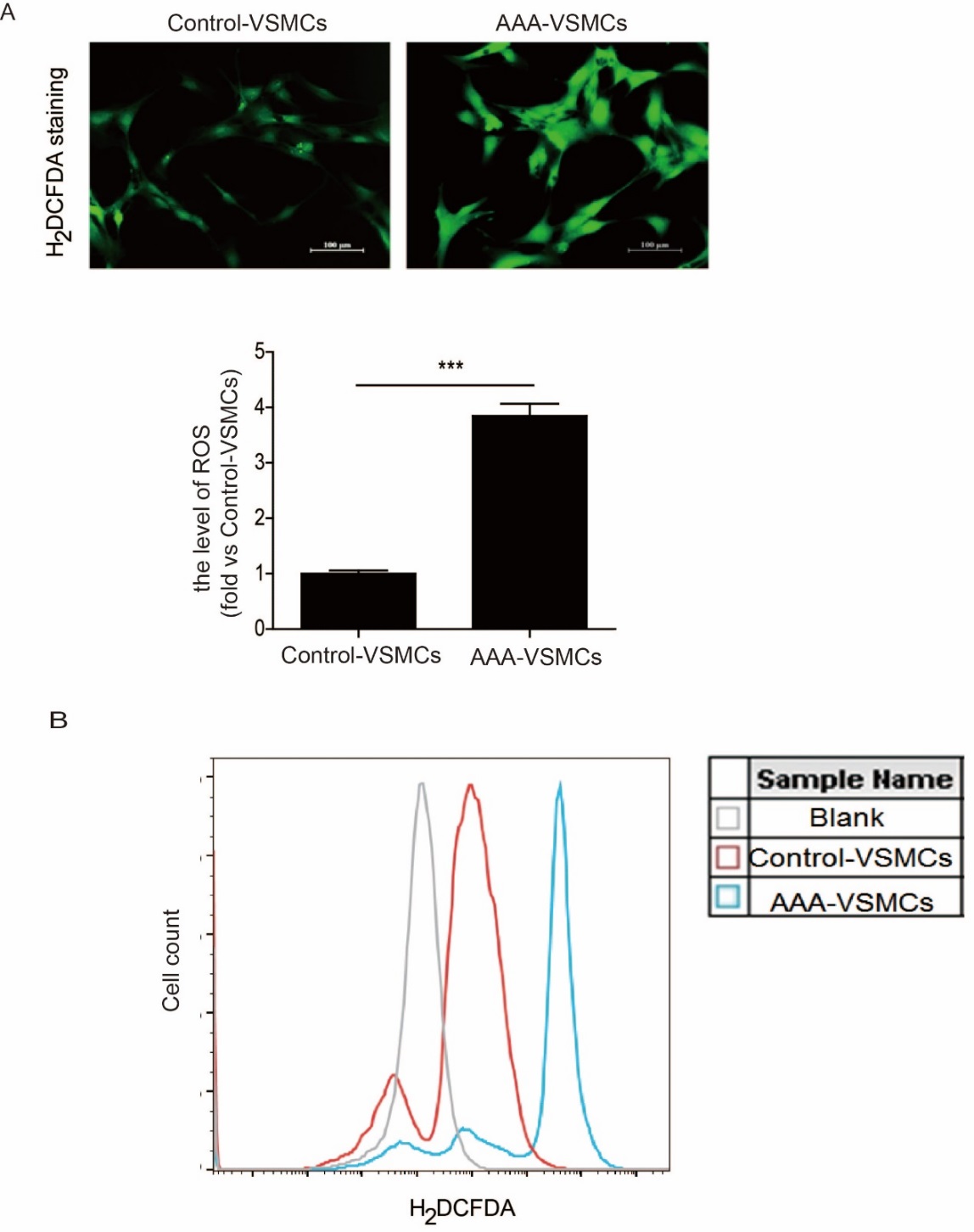


**Supplemental Figure 4. Overexpressed Sirt1 rescued Ang II-induced VSMC senescence and inhibited ROS generation**

(A) Representative images of SA-β-gal staining and quantitative analysis of SA-β-gal-positive cells in Ang II-treated or Ang II +Lenti-Sirt1-treated control-VSMCs. (B) Representative images of H_2_DCFDA staining and quantitative analysis of ROS generation in Ang II-treated or Ang II +Lenti-Sirt1-treated control-VSMCs. ****p<0.001.*

**
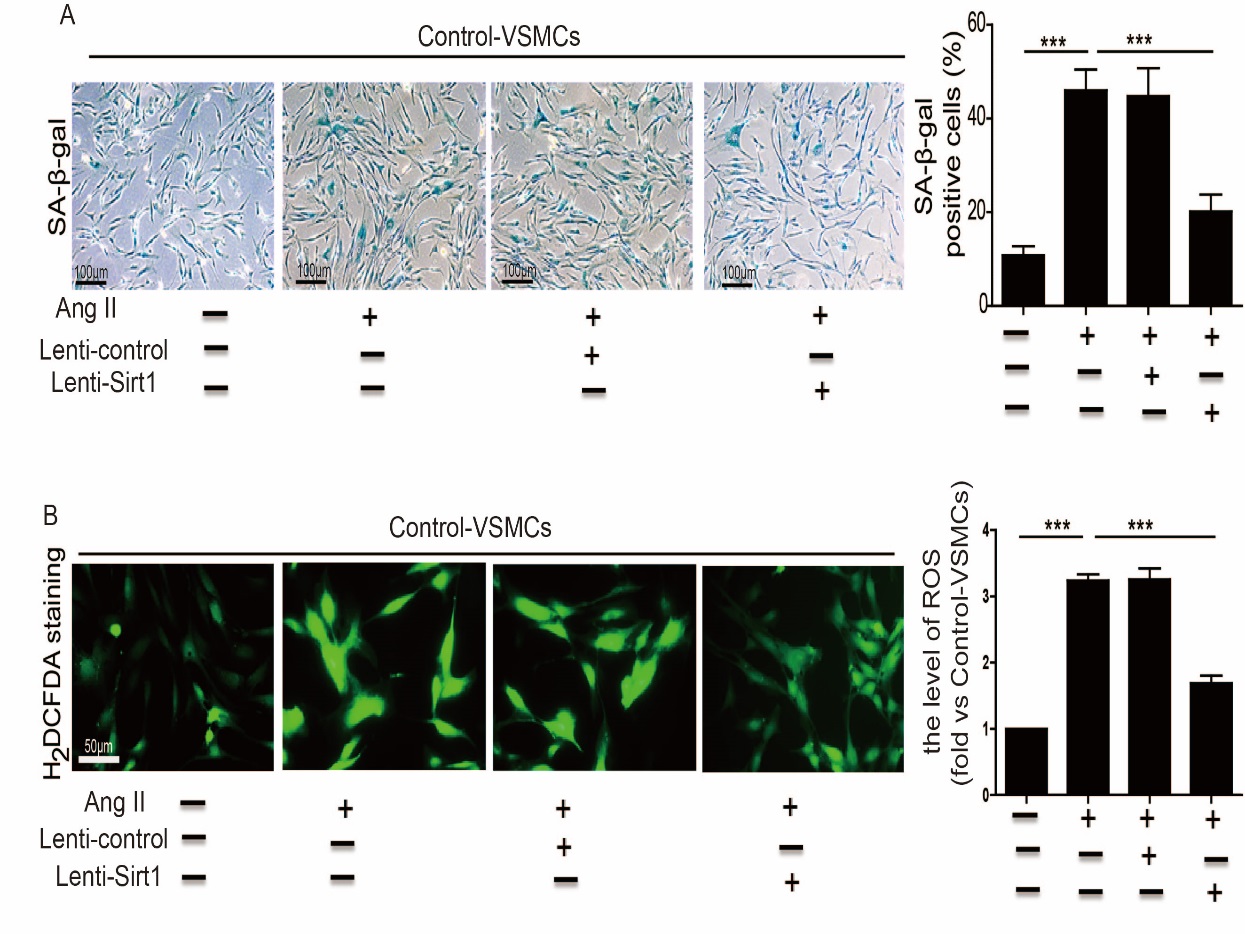
**

**Supplemental Figure 5. The expression level of inflammatory factors in AAA tissues.**

(A) Concentration of IL-6 in AAA tissues and control tissues. (B) Concentration of TNF-α in AAA tissues and control tissues. ***p<0.01, ***p<0.001.*


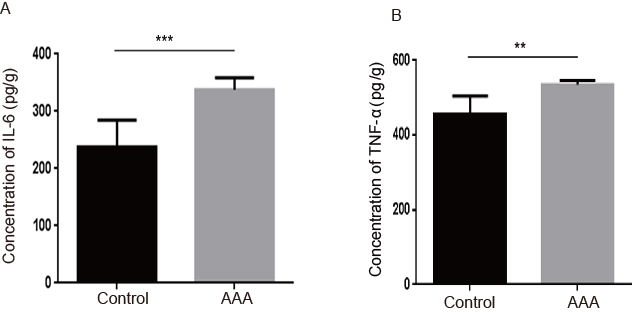

Supplement: Supplementary file 1 — Fig S1‐S5 [file JCMM-25-6056-s001.docx]
